# Enhancing Effect of Glycerol on the Tensile Properties of *Bombyx mori* Cocoon Sericin Films

**DOI:** 10.3390/ijms12053170

**Published:** 2011-05-16

**Authors:** Haiping Zhang, Lianxia Deng, Mingying Yang, Sijia Min, Lei Yang, Liangjun Zhu

**Affiliations:** Institute of Applied Bioresources, College of Animal Sciences, Zhejiang University, Hangzhou 310029, China; E-Mails: hpzhang@zju.edu.cn (H.Z.); denglianxia2008@163.com (L.D.); yangm@zju.edu.cn (M.Y.); minsj@zju.edu.cn (S.M.); yanglei164@163.com (L.Y.)

**Keywords:** glycerol, sericin, tensile strength, ATR-FTIR, secondary structure

## Abstract

An environmental physical method described herein was developed to improve the tensile properties of *Bombyx mori* cocoon sericin films, by using the plasticizer of glycerol, which has a nontoxic effect compared with other chemical crosslinkers. The changes in the tensile characteristics and the structure of glycerolated (0–40 wt% of glycerol) sericin films were investigated. Sericin films, both in dry and wet states, showed enhanced tensile properties, which might be regulated by the addition of different concentrations of glycerol. The introduction of glycerol results in the higher amorphous structure in sericin films as evidenced by analysis of attenuated total reflection Fourier transform infrared (ATR-FTIR) spectra, thermogravimetry (TGA) and differential scanning calorimetry (DSC) curves. Scanning Electron Microscopy (SEM) observation revealed that glycerol was homogeneously blended with sericin molecules when its content was 10 wt%, while a small amount of redundant glycerol emerged on the surface of sericin films when its content was increased to 20 wt% or higher. Our results suggest that the introduction of glycerol is a novel nontoxic strategy which can improve the mechanical features of sericin-based materials and subsequently promote the feasibility of its application in tissue engineering.

## 1. Introduction

Tissue engineering is an increasingly popular approach for impaired tissues and organs therapy. In the course of tissue engineering, functional materials with excellent biocompatible and biodegradable properties are required to function as the extracellular matrix. Naturally derived polymers have been regarded as preferable alternatives for potential tissue engineering biomaterials [1,2]. Proteins are suitable natural polymers, widely distributed in nature and can be effectively processed into various shapes like film, hydrogel and fiber. Collagen [3], fibroin [4,5], gelatin [6] are the most commonly utilized proteins for tissue engineering application. Biomaterials based on these proteins still cannot satisfy all the tissue engineering requirements due to a few problems in the biocompatibility, the mechanical properties and the degradation ratio. Therefore, it is necessary to develop novel materials to address the tissue engineering requirements.

Sericin is a global protein, synthesized exclusively in the middle silk glands of silkworm, *Bombyx mori* and contributes 20–30 wt% of the total silk cocoon weight. A large amount of polar amino acids such as 32% serine and 17% aspartic acid renders sericin its higher hydrophilic property and processing ability. Therefore, sericin has been proposed as a promising natural resource for developing protein-based tissue engineering biomaterials. In order to realize the application of sericin in the field of tissue engineering, many researches including promotion of wound healing [7,8], hydroxyapatite crystals induction [9–11], drugs immobilization [12–14] and enhancement of cells attachment and proliferation [15,16] have been carried out.

Film-shaped biomaterials have been suggested as one of the important formulations in the field of tissue engineering [17,18]. However, the cast dry films based on silk cocoon sericin show fragile tensile properties. This leads to the difficulty of obtaining integrated sericin formulations and the inconvenience of its practical application. To avoid such hurdles, chemical cross-linkers, such as polyethylene glycol diglycidyl ether (PEG-DE) [19] and glutaraldehyde and dimethylolurea (DMU) [20], were usually used to improve the tensile properties of sericin-based films. However, the chemical cross-linker reagents can result in toxicity problems and lower biocompatibility. Consequently, novel strategies without any toxicity for preparation of sericin-based film are urgently required.

In this study, the flexible sericin films were developed by blending glycerol with sericin. The elastic modulus, tensile strength and elongation at break of sericin films with and without glycerol were characterized to elucidate the effect of glycerol on the tensile properties of sericin films. Fourier transform infrared (FTIR), thermogravimetry (TGA), differential scanning calorimetry (DSC) and Scanning Electron Microscopy (SEM) determination were conducted to analyze the structural changes of sericin films.

## 2. Results and Discussion

### 2.1. Secondary Structure Transition

**Figure 1.**
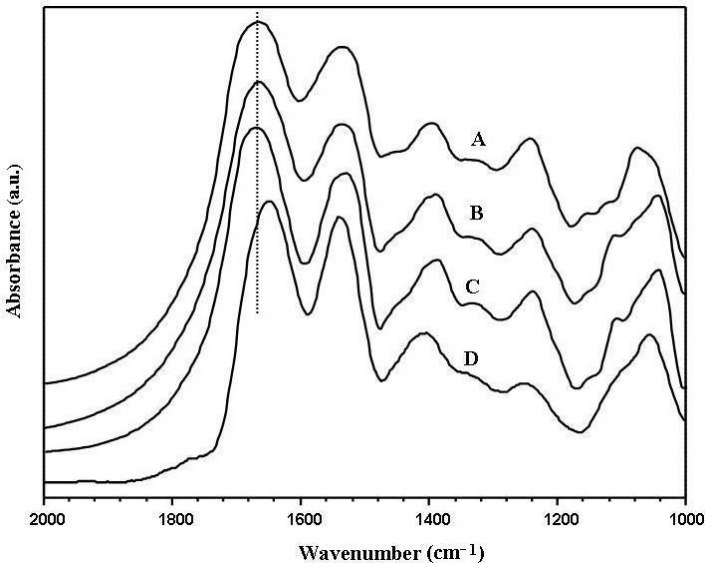
Attenuated total reflection Fourier transform infrared (ATR-FTIR) spectra of sericin films (one experimental spectrum is shown as the representative for each sericin film type): (**A**) sericin film; (**B**) sericin film with 10 wt% glycerol; (**C**) sericin film with 20 wt% glycerol; (**D**) sericin film with 30 wt% glycerol.

The secondary structure of sericin film and glycerol blended sericin films were characterized by attenuated total reflection Fourier transform infrared (ATR-FTIR). Figure 1 shows one of the ATR-FTIR original experimental spectra for sericin films with different content of glycerol as the representative. The amide I band at 1600–1700 cm^−1^ represents the C=O stretching vibration of the amide group as the most sensitive region to protein secondary structure and has been widely used to identify the secondary structure change of proteins [21,22]. As shown in Figure 1, the position of the maxima in the amide I band of sericin film without glycerol was observed at 1663 cm^−1^, and that of sericin films with 10 wt%, 20 wt% and 30 wt% glycerol was at 1663 cm^−1^, 1664 cm^−1^ and 1645 cm^−1^, respectively. Infrared absorption observed at about 1663 and 1645 cm^−1^ is usually assigned as the turn and the random coil, respectively [23]. Consequently, the position of the maxima suggested that sericin films with 10 wt% and 20 wt% glycerol shows the main structure of turns similar to sericin film without glycerol. While sericin film with 30 wt% glycerol adopts mainly random coil.

**Figure 2.**
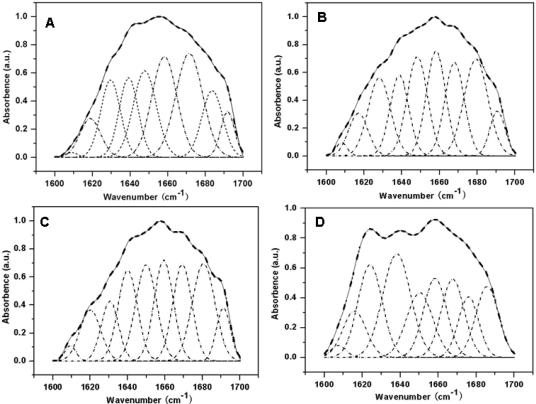
Curve-fitted spectra of various sericin films at amide I band between 1600–1700 cm^−1^: (**A**) sericin film; (**B**) sericin film with 10 wt% glycerol; (**C**) sericin film with 20 wt% glycerol; (**D**) sericin film with 30 wt% g 0.39″ lycerol. The broken lines represent the Gaussian fitted curves and the solid lines represent the deconvolution spectra of amide I band.

**Figure 3.**
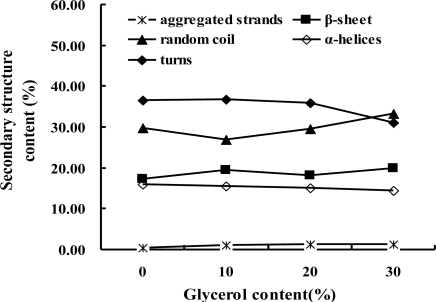
The secondary structure components distribution of sericin films with different content of glycerol.

To further clarify the effect of glycerol on the secondary structure of sericin film, the quantitative analysis on amide I band of various sericin films was carried out using the Fourier Self Deconvolution (FSD) fitting method. As shown in Figure 2, each sericin film at amide I region were fitted to nine single bands calculated from the second derivative spectra and the area of the fitted single peaks are shown in Table 1. The overlapped nine single bands to the secondary structure were assigned as follows and according to the previous studies [24]: band at 1608 cm^−1^ as the structure of aggregated strands, 1617 cm^−1^ and 1627 cm^−1^ as β-sheet, 1638 cm^−1^ and 1648 cm^−1^ as random coil, 1658 cm^−1^ as α-helices and 1668 cm^−1^, 1679 cm^−1^ and 1690cm^−1^ as turns. The percent content of various secondary structure components in sericin films were calculated as the data in Table 1 and their distribution were shown in Figure 3. It is observed that sericin film, 10 wt% and 20% glycerol blended sericin films predominantly exhibit turns with the content of about 37%, 37% and 36%, respectively. However, the content of turns in sericin film with 30 wt% was about 31% and less than the content of random coil as about 33%. This result indicated that the main secondary structure of sericin film changed to random coil when glycerol content was increased to 30%, which was identical to the estimated structure from the maxima position in the amide I band of the ATR-FTIR original spectra. When glycerol content was 10 wt%, the content of random coil structure decreased from about 30% to 27% and the content of β-sheet increased from about 17% to 20%, due to the physical interaction such as hydrogen bond between sericin and small glycerol molecules. On further increasing the content of glycerol, the content of random coil was greatly increased and the turns decreased, as a result of the plasticizer function of many glycerol molecules by reducing the interaction of sericin molecules.

### 2.2. Thermal Characterization

**Figure 4.**
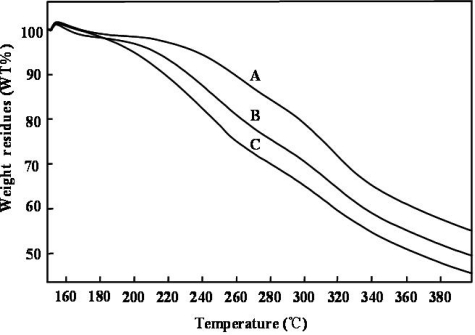
TGA thermographs of various sericin films: (**A**) sericin film; (**B**) sericin film with 10 wt% glycerol; (**C**) sericin film with 20 wt% glycerol.

To further investigate the effect of glycerol on the sericin films structure, the thermal behaviors of sericin films with and without glycerol were determined by TGA and DSC analysis. Figure 4 shows TGA thermographs of sericin films before and after adding the glycerol, which describe the thermal decomposition characteristics of these films. These results indicated that sericin films with glycerol decomposed more quickly than those without glycerol. In addition, sericin films with 20 wt% glycerol decomposed more quickly compared with those with 10 wt% glycerol, suggesting that glycerol can accelerate the decomposition of sericin films.

**Figure 5.**
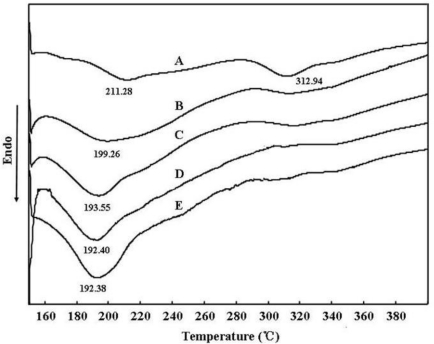
DSC curves of various sericin films: (**A**) sericin film; (**B**) sericin film with 10 wt% glycerol; (**C**) sericin film with 20 wt% glycerol; (**D**) sericin film with 30 wt% glycerol; (**E**) sericin film with 40 wt% glycerol.

More details about the thermal properties of sericin films can be revealed from the DSC curves (Figure 5). As shown in Figure 4, sericin films have two endothermic peaks at 211.28 °C and 312.94 °C, respectively, which are attributed to the thermal decomposition of the sericin compositions [25]. The first endothermic peak is denoted as the amorphous structure and the second peak is assigned to a more stable structure like crystallinity in sericin films [26]. DSC curves of sericin films with glycerol show that the first endothermic peak decreased with the glycerol content and the second peak disappeared gradually. This implied that the crystallinity of sericin film decreased with the increase of glycerol and consequently thermal stability decreased. This result coincides with the TGA analysis.

### 2.3. Microscopic Structure Analysis

**Figure 6.**
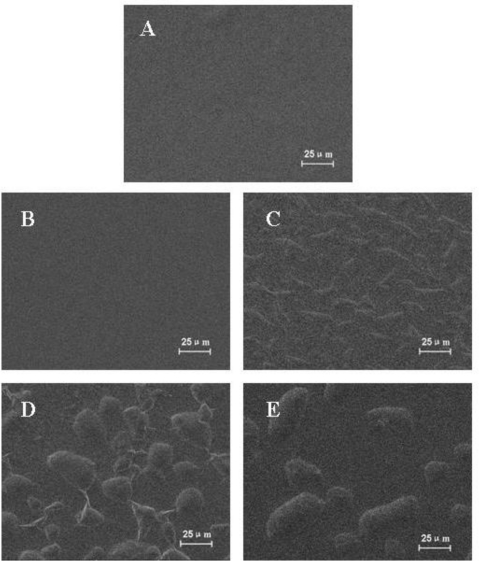
SEM images of various sericin films: (**A**) sericin film; (**B**) sericin film with 10 wt% glycerol; (**C**) sericin film with 20 wt% glycerol; (**D**) sericin film with 30 wt% glycerol; (**E**) sericin film with 40 wt% glycerol.

The morphology change of sericin films after the addition of glycerol is shown in Figure 6. Both sericin films without glycerol and with 10 wt% glycerol showed smooth surface (Figure 6A,B), which implied that glycerol is homogeneously distributed in the films with 10 wt% glycerol. However, when the glycerol content was increased more than 20 wt%, many protuberances were observed on the surface of these films (Figure 6C–E) due to the aggregation of the redundant glycerol molecules. In the case of sericin films with 20 wt% glycerol, the length of protuberances was 15–25 μm and the width was 3–7 μm. When the content of glycerol in the film was increased to 30 wt%, the length and width increased to 17–44 μm and 11–30μm, respectively. The protuberances increased to 27–82 μm in the length and 16–39 μm in the width when the content of glycerol is 40 wt%. The number of protuberances in sericin films with 20 wt%, 30 wt% and 40 wt% glycerol were about 233, 97 and 66, respectively, in the area of 590 μm × 350 μm. These results indicated that the size of the protuberances increased with the content of glycerol while their numbers decreased.

### 2.4. Tensile Properties

To examine the effect of glycerol on the physical properties of sericin films, the tensile properties of sericin films with and without glycerol were determined and the results are shown in Table 2 (in dry state) and Table 3 (in wet state). In comparing the data of the two tables, it was concluded that sericin films in wet state showed extremely lower elastic modulus and tensile strength than those in dry state. The distinguished difference in mechanical properties between sericin films in the two states is mainly due to the higher hydrophilic property of sericin molecules, which is also common in other functional materials [22,27].

Sericin films both in dry state and wet state showed unsatisfactory mechanical properties for practical application due to the striking brittleness and the weak strength, respectively. Therefore, it is important to improve the flexibility of dry sericin film and the strength of wet sericin film. From the two tables, it was found that the elongation at break of sericin films both in dry and wet states was greatly increased with the addition of glycerol. This was due to the plasticizer property of glycerol and has been commonly applied in other materials for higher flexibility [22,28,29]. In addition, sericin film with 10 wt% glycerol showed higher tensile strength than that of sericin film without glycerol, due to the structural change of sericin film as shown in FTIR. These results indicated that glycerol can be used to improve the mechanical properties of sericin film.

By comparing the groups of sericin films with 10 wt%, 20 wt%, 30 wt% and 40 wt% glycerol, it was observed that the tensile properties of sericin film both in dry and wet states was changed with the content of glycerol. Table 2 showed that the elastic modulus and the elongation at break of sericin film in dry state decreased and increased with the content of glycerol, respectively, indicating that the flexibility of sericin film was improved with the increase of glycerol. However, the tensile strength was decreased with the glycerol content which was caused by the higher amorphous structure as indicated in the FTIR, DSC and TGA results. From Table 3, it was found that the flexibility of sericin film in wet state was also increased with the glycerol content. The tensile strength of sericin film in wet state was increased when the glycerol content was increased from 10 wt% to 20 wt%, and then basically unchanged with higher glycerol. This phenomenon was presumably induced by the physical interaction among sericin, glycerol and water molecules. When glycerol content was less than 20 wt%, the physical interaction among sericin, glycerol and water molecules was increased with the glycerol content. When the glycerol content was higher, the physical interaction among the three molecules reached the maximum level and glycerol was redundant as shown in SEM images which can dissolve in water. The results need to be further confirmed through future study on the structure of glycerolated sericin films in wet state.

## 3. Experimental Section

### 3.1. Materials

Cocoon shells of *Bombyx mori* silkworm were provided by the Institute of Huzhou Cocoon Testing (P. R. China). Glycerol was purchased from Sigma-Aldrich. Ultrapure water was prepared through the Milli-Q plus system (Millipore Corporation, USA).

### 3.2. Sericin Solution Extraction

Sericin solution was extracted from *Bombyx mori* cocoon as our previous description [30]. Briefly, the cocoon shells were cut into pieces (about 1 cm^2^) and then immersed in boiling water (the weight ratio of sericin to water is 1:30) for about 30 min to obtain the sericin solution. Then the solution was discolored with active carbon after filtrating with gauze. Finally, the discolored solution was concentrated to 8 wt%.

### 3.3. Preparation of Sericin Films with and without Glycerol

Sericin films without glycerol were prepared by casting sericin solution into the plastic petri dishes and drying at 65 °C for about several hours. After dried, the films were peeled gently from the dish and stored in desiccators for characterization. The glycerol-blended sericin films were prepared by drying the mixture solution of sericin and glycerol at 65 °C. The mixture solution was obtained by slowly injecting glycerol into the sericin solution with continuous magnetic stirring. The weight ratios of glycerol to sericin were regulated as 10, 20, 30 and 40 wt%. The thickness of all films was controlled to 80 ±5 μm.

### 3.4. Tensile Properties

Tensile tests were performed on a mechanical testing machine (AGS-J, Shimadzu, Japan) equipped with a 50 N load cell for the dry test and a 5 N load cell for the wet test. Film samples were cut into strips with the width of 0.5 cm and the length of 5 cm for testing. The length between two gauges was controlled at 1.5 cm. For dry test, films were tested after loaded onto the tester for 2 days at 25 °C and 50% R.H. For wet test, samples were soaked in the deionized water for 5 min before loaded onto the tester. The elastic modulus, the tensile strength and the elongation at break of the films were calculated from the stress-strain curves. Data represent the average ±SD (*n* = 3).

### 3.5. Attenuated Total Reflection-Fourier Transform Infrared Analysis (ATR-FTIR)

ATR-FTIR absorption spectra of sericin films at the wavenumber range of 1000–2000 cm^−1^ were determined with a Fourier Transform Infrared Instrument (FTIR-8400 S, Shimadazu, Japan) equipped with an Attenuated Total Reflection attachment (ATR-8000, Shimadazu, Japan) in the reflection mode. Samples were cut into 3 × 3 cm^2^ and fixed with KRS-5 crystal prism for determination. For each measurement, scanning was repeated 128 times with a resolution of 4 cm^−1^. Measurements were made on three samples for each type.

The quantitative analysis on the structural characteristics of sericin films was carried out at the amide I band between 1600–1700 cm^−1^ through the peak-fitting method. Prior to fitting amide I band, the baseline correction with a straight line between 1600–1700 cm^−1^ and the nine-point smoothing of ATR-FTIR spectra were performed. The second derivative spectra for calculating the number and position of the overlapped single bands and the deconvolution spectra for fitting were obtained. All the pretreatment of spectra were performed with IRsolution software (Shimadazu, Japan). The curve fitting process was iteratively performed until the parameter of Chi^2/Dof reached to the minimum value with the multi-peaks Gaussian fitting by Origin software. The relative area of the overlapped single bands are used for estimating the proportion of secondary structures such as random coil, α-helices, β-sheet and turns. The single bands of sericin films were assigned to the corresponding secondary structures according to previous studies [24]: 1605–1615 cm^−1^, aggregated strands; 1616–1637 cm^−1^, β-sheet; 1638–1655 cm^−1^, random coil; 1656–1662 cm^−1^; α-helices; 1663–1695 cm^−1^, turns.

### 3.6. Thermogravimetric Analysis (TGA)

Thermogravimetric analysis of sericin films was conducted by using a TGA instrument (TGA/SDTA851, Mettler Toledo, Switzerland). The thermograms were obtained under a nitrogen atmosphere (50 mL/min) at a uniform heating rate of 20 °C/min in the temperature range of 150–400 °C.

### 3.7. Differential Scanning Calorimetry (DSC)

DSC curves of sericin films were measured with a differential scanning calorimetry instrument (DSC 822e, Mettler Toledo, Switzerland) between the temperature range of 150–400 °C. The heating rate for test was kept at 10 °C/min and the nitrogen gas flow rate was controlled at 50 mL/min.

### 3.8. Scanning Electron Microscopy (SEM)

Sericin films samples were sputter coated with gold ion sputtering instrument (IB-5, Eiko, Japan) for 5 min and then glued on the surface of sample stage with conductive tape. The morphology of sericin films was observed with a scanning electron microscope (XL30-ESEM, Philips, Netherlands).

## 4. Conclusions

*B. mori* sericin films with different content of glycerol were prepared. The structure stability of sericin film decreased with the content of glycerol as shown in the FTIR, DSC and TGA results. SEM results revealed that glycerol can homogeneously blend with sericin when its content was 10 wt% and the redundant glycerol aggregated on the surface of sericin film. The tensile strength of sericin film both in dry and wet states increased when the glycerol content was 10 wt%. The flexibility of sericin film in dry state was greatly increased with the addition of glycerol which can advance the application of sericin film. Therefore, glycerol is an effective green substance to improve the mechanical properties of sericin films.

## Figures and Tables

**Table 1.** The relative proportion (%) of the secondary structures calculated from the areas of the fitted overlapped peaks of sericin films with different content of glycerol.

**Peaks**	**1608**	**1617**	**1627**	**1638**	**1648**	**1658**	**1668**	**1679**	**1690**

**Secondary Structure**	**Aggregated Strands**	**β-Sheet**		**Random Coil**		**α-Helices**	**Turns**		
0% Gly	0.36 ± 0.05	17.19 ± 0.3		29.86 ± 2.4		15.91 ± 2.2	36.67 ± 0.1		
10% Gly	1.15 ± 0.01	19.58 ± 0.2		26.99 ± 0.8		15.46 ± 1.2	36.81 ± 0.3		
20% Gly	1.35 ± 0.02	18.08 ± 1.4		29.53 ± 0.7		15.16 ± 1.1	35.88 ± 1.7		
30% Gly	1.25 ± 0.01	20.01 ± 2.0		33.28 ± 1.2		14.37 ± 1.3	31.09 ± 0.5		

**Table 2.** Tensile properties of sericin films with different glycerol content in dry state.

**Glycerol Content (%)**	**Elastic Modulus (MPa)**	**Elongation at Break (%)**	**Tensile Strength (MPa)**
0	600.53 ±76.30	0.73 ±0.10	13.72 ±0.30
10	391.40 ±52.73	140.62 ±35.66	17.35 ±0.78
20	288.21 ±46.36	172.50 ±43.63	14.38 ±2.20
30	77.06 ±7.28	250.40 ±59.30	13.53 ±2.07
40	57.31 ±8.28	354.37 ±35.72	8.19 ±1.06

**Table 3.** Tensile properties of sericin films with different glycerol content in wet state.

**Glycerol Content (%)**	**Elastic Modulus (MPa)**	**Elongation at Break (%)**	**Tensile Strength (MPa)**
0	0.64 ±0.09	53.58 ±8.69	0.21 ±0.05
10	4.08 ±0.53	130.37 ±29.67	0.73 ±0.09
20	3.70 ±0.45	168.24 ±31.47	1.15 ±0.07
30	2.68 ±0.32	244.67 ±51.70	1.12 ±0.08
40	2.26 ±0.26	271.33 ±42.51	1.18 ±0.08
